# A New Cathepsin D Targeting Drug Delivery System Based on Immunoliposomes Functionalized with Lipidated Pepstatin A

**DOI:** 10.3390/pharmaceutics15102464

**Published:** 2023-10-14

**Authors:** Andreja Kozak, Georgy Mikhaylov, Pavlo Khodakivskyi, Elena Goun, Boris Turk, Olga Vasiljeva

**Affiliations:** 1 Department of Biochemistry and Molecular and Structural Biology, Jozef Stefan Institute, 1000 Ljubljana, Slovenia; 2 SwissLumix SARL, 1015 Lausanne, Switzerland; 3 Department of Chemistry, University of Missouri-Columbia, Columbia, MO 65211, USA; 4 Faculty of Chemistry and Chemical Technology, University of Ljubljana, 1000 Ljubljana, Slovenia

**Keywords:** tumor targeting, immunoliposomes, breast cancer, protease, cathepsin D, pepstatin A

## Abstract

Cathepsin D is an aspartic protease and one of the most abundant proteases. It is overexpressed in many cancers and plays an important role in tumor development, progression, and metastasis. While it is a physiologically intracellular protein, cathepsin D is secreted into the extracellular matrix under pathological conditions, making it an appealing target for drug delivery systems. Here, we present the development and evaluation of a new delivery system for tumor targeting based on immunoliposomes functionalized with pepstatin A—a natural peptide inhibitor of cathepsin D. A lipid tail was added to pepstatin A, enabling its incorporation into the liposomal lipid bilayer. The successful targeting of cathepsin D was confirmed using recombinant cathepsin D and in tumor cell lines, showing the feasibility of this targeting approach and its potential for in vivo use in theragnostic applications.

## 1. Introduction

Cancer is one of the leading causes of death worldwide, and chemotherapy is one of the principal strategies used to combat it. The need to minimize the side effects of chemotherapeutic drugs and to increase their therapeutic windows has led to the development of targeted therapies, which enable the localization or activation of a drug at the tumor site [1,2,3]. The tumor microenvironment (TME) is a complex system consisting of various cancerous and non-cancerous cells and the extracellular matrix, the latter of which supports tumor growth and proliferation [4,5]. Proteases are a major group of proteins that contribute to the overall tumor-promoting effects of the TME. Most cancer-associated proteases are extracellular and can be secreted into the TME by tumor cells, cancer-associated immune cells, or even healthy cells, such as osteoclasts, adipocytes, or fibroblasts. Among the most researched proteases in the TME are matrix metalloproteinases (MMPs) and cysteine cathepsins [6,7]. In contrast to MMPs, cysteine cathepsins are mainly localized intracellularly in the endosomal/lysosomal compartment; however, they are known to be present extracellularly in pathological conditions and thus could be considered safer targets. Recently, targeted drug delivery systems based on inhibitors or substrates of cysteine cathepsins have been developed for therapeutic and theragnostic applications [8,9,10]. However, aspartic cathepsins are also known to be important in various stages of tumor formation and progression. Specifically, cathepsin D has been the focus of many studies, elucidating its role in cancer and highlighting its potential as a biomarker or drug target [11], although research utilizing this potential remains limited.

Cathepsin D is a ubiquitously expressed lysosomal aspartic protease that has traditionally been associated with general protein turnover in lysosomes [12,13]. Notably, cathepsin D is overexpressed in many cancers and has been associated with a poor prognosis [11,13,14,15,16,17,18]. Cathepsin D is excreted into the TME, predominantly by tumor cells, where it can be activated and sustained due to the low pH in the TME [17]. The proteolytic activity of cathepsin D leads to pro-tumor factor activation and extracellular matrix cleavage [14,15,18]. In addition to its proteolytic activity, cathepsin D promotes tumor growth through non-proteolytic mechanisms involving the activation of kinase pathways, namely, the ERK1/2 and AKT pathways [16,19]. Its extracellular localization and tumor overexpression, therefore, make it an interesting target for tumor-targeting drug delivery strategies.

Among drug delivery nanocarriers, liposomes are among the most widely used systems due to their several advantages [20,21]. First, their lipid bilayer membrane permits the incorporation of hydrophobic compounds, while encapsulating hydrophilic agents into the aqueous center is also possible. Second, their surfaces can be readily functionalized by addition of (i) polyethylene glycol (PEG), which improves liposome stability and enhances their circulation time in the blood by reducing in vivo opsonization; (ii) various targeting molecules, which can be coupled with the liposome surface, such as antibodies, peptides, or carbohydrates; or (iii) imaging agents, which can be incorporated into their lipid bilayer or encapsulated within, enabling the visualization of liposomes, which can be used for theragnostic purposes [22]. Importantly, liposomes are biocompatible, non-toxic, non-immunogenic, and biodegradable, making them ideal therapeutics, diagnostics, or vaccine carriers [23,24,25,26]. Some liposomal formulations have already been approved for clinical use in addition to their extensive utilization in preclinical research [27]. Recently, Moderna and Pfizer-BioNTech both employed lipid nanoparticles (LNPs) for mRNA-based vaccines against the SARS-CoV-2 virus [28,29]. LNPs are similar to liposomes but have a more complex structure with several inner layers of membranes. They are composed of ionizable lipids, which are positively charged at low pH levels in order to bind mRNA and neutral at physiological pH levels to prevent toxicity [30].

**Figure 1:**
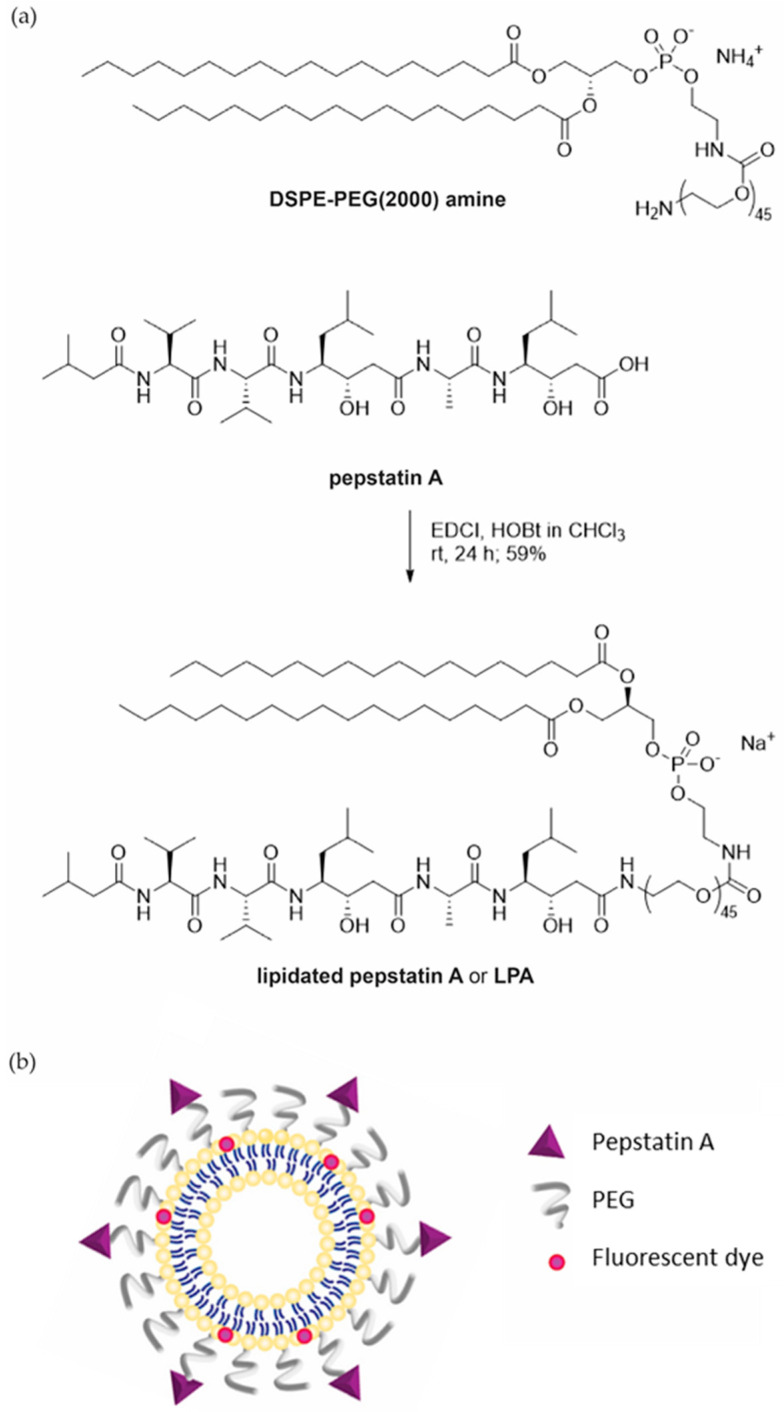
(**a**) Synthesis of the lipidated pepstatin A (LPA): a PEGylated lipid was conjugated to the cathepsin D inhibitor pepstatin A through the reaction between a corresponding amine and carboxyl groups using standard coupling reagents. (**b**) A schematic representation of the pepstatin A liposomal system (PepA-L): lipidated pepstatin A was inserted into the lipid bilayer of liposomes, which also contained a fluorescent dye (rhodamine).

Here, we present the development of a liposomal-based delivery system for targeting cathepsin D using its peptide inhibitor—pepstatin A (Figure 1b). Pepstatin A is a well-established potent inhibitor of microbial origin. It is a hexapeptide, and it contains an unusual amino acid—statine [31]. Pepstatin A inhibits several aspartic proteases, among which is cathepsin D, by occupying their substrate-binding clefts [32]. We exploited its high affinity for cathepsin D, thus employing it as a targeting moiety on the surfaces of liposomes, forming so-called immunoliposomes, and demonstrated its ability to bind to tumor cell surfaces, thus confirming its potential for theragnostic applications as a targeted drug delivery system.

## 2. Materials and Methods

### 2.1. Synthesis of the Lipidated Pepstatin A

1,2-distearoyl-sn-glycero-3-phosphoethanolamine-N-[amino(polyethylene glycol)-2000] (DSPE-PEG(2000) amine) (Avanti Polar Lipids, Alabaster, AL, USA, 14.0 mg, 5 µmol) was added to a mixture of pepstatin A (Sigma-Aldrich, St. Louis, MO, USA, 6.9 mg, 10 µmol), EDCI (Acros Organics, Geel, Belgium, 3.8 mg, 20 µmol), HOBt (Sigma-Aldrich, 2.0 mg, 15 µmol), and CHCl_3_ (1.5 mL), which was stirred at 0 °C. The reaction mixture was then left to warm up to approximately 21–23 °C, and this was followed by stirring for 24 h (monitored via UPLC, Waters AQUITY H-Class, water-acetonitrile gradient, RP C18 column). The reaction mixture was then diluted with CHCl_3_ (30 mL) and washed three times with 5% NaHCO_3(aq)_ and brine. The organic layer was dried over anhydrous Na_2_SO_4_ and filtered, and the solvent was evaporated under reduced pressure. The solid obtained was dissolved in acetonitrile/water (1:1 *v*/*v*) and lyophilized to obtain lipidated pepstatin A as a colorless powder.

### 2.2. Liposome Preparation

Liposomes were prepared from chicken egg L-a-phosphatidylcholine (63 mol %) (Avanti Polar Lipids), cholesterol (33 mol %) (Sigma), and MeO-PEG (4 mol %) (Avanti Polar Lipids). For the pepstatin A functionalized liposomes (PepA-L), methoxy-PEG was replaced with lipidated PEG-pepstatin A (LPA). The total lipid concentration was 3 mM. Lissamine rhodamine B (0.1 mol %) (Avanti Polar Lipids) was added to the lipid mixture to prepare rhodamine liposomes (Rho-L and PepA-Rho-L). The organic solvent was evaporated in an Eppendorf Concentrator 5301 (Eppendorf, Hamburg, Germany). The dry lipid film formed was then hydrated in degassed 100 mM sodium phosphate buffer (pH 7.0, 150 mM NaCl). To generate nanosized unilamellar bilayer liposomes, the multilamellar formed vesicles were extruded using a mini-extruder (Avanti Polar Lipids), fitted with a polycarbonate membrane with a pore size of 400 nm, and then extruded through a 100 nm pore size membrane.

### 2.3. Inhibition of Cathepsin D

Cathepsin D (R&D Systems, Minneapolis, MN, USA) was assayed in 0.1 M acetate buffer (pH 3.5) using z-GLPL-AMC (Enzo Life Systems, New York, NY, USA) as a substrate. The same approach was also used to analyze the inhibition of cathepsin D by pepstatin A and the liposomal system.

### 2.4. Cell Culture

4T1—a highly aggressive and metastatic triple-negative breast cancer cell line; A549—less aggressive non-small-cell lung carcinoma cells; B16F10—melanoma cells, frequently utilized in metastasis research; CaCo-2—a heterogenous culture of colon adenocarcinoma cells; MDA-MB-231—an aggressive type of triple-negative breast cancer cells, often used to model late-stage breast cancer; SK-BR-3—a HER2-overexpressing breast cancer cell line; and RAW 264.7—a macrophage-like cell line, established from a leukemia-induced mouse tumor, were obtained from the American Type Culture Collection as ATCC CRL-2539, ATCC CRM-CCL-185, ATCC CCL-185, ATCC CRL-6475, ATCC CRM-HTB-26, ATCC HTB-30, and ATCC SC-6003, respectively. RAW 264.7 are often used as a macrophage model; they respond to LPS stimulation and are able to produce nitric oxide. Primary tumor cells from a transgenic PyMT mouse breast cancer model, which closely mimics human breast cancer progression, were isolated and grown as described previously [33]. Cells were grown in DMEM (Sigma) medium containing 10% FBS, 1% P/S, and 1% Glutamax.

### 2.5. Cell Membrane Association Assay

Cells (40,000 per well) were seeded into black 96-well plates with transparent bottoms (Falcon) and grown overnight at 37 °C and 5% CO_2_. The medium was replaced with DMEM containing 50 mM of HEPES (Sigma). Cells were then incubated at 4 °C for 1 h to block the endocytosis process; all additional steps were also performed at 4 °C. After being washed two times with PBS, cells were incubated for 1 h in PBS containing rhodamine-liposomes (Rho-L and PepA-Rho-L). Next, cells were washed with PBS, and a plate reader (TECAN, Männedorf, Switzerland) was used to measure fluorescence intensity at nine locations in each well.

### 2.6. Immunoblotting of Cathepsin D in Cell Lysates

Cell lysates were obtained by incubating cells in RIPA lysing buffer. Cell lysates were then boiled in SDS-PAGE loading buffer, followed by SDS-PAGE and immunoblotting using anti-human cathepsin D antibodies (produced in our laboratory [34], dilution 1:1000) or anti-mouse cathepsin D antibodies (Abcam, Cambridge, UK, ab75852, dilution 1:1000). For internal control, anti-GAPDH antibodies (Cell Signaling Technology, Danvers, MA, USA, 2118, dilution 1:1000) were used. Membranes were incubated in primary antibody solution overnight at 4 °C, followed by washing and incubation with secondary antibodies, namely, HRP-goat anti-rabbit IgG (H+L) (Jackson Immunoresearch, West Grove, PA, USA, 111-035-045, dilution 1:5000) for human cathepsin D and GAPDH and HRP-goat anti-mouse IgG, IgM (H+L) (Jackson Immunoresearch, 115-035-068, dilution 1:5000) for mouse cathepsin D.

### 2.7. Immunocytochemistry

Cells were seeded onto glass coverslips and fixed with 4% PFA for 30 min at approximately 21–23 °C. Cells were then labeled with anti-cathepsin D antibodies, followed by Alexa Fluor 555 anti-rabbit IgG antibodies. Coverslips were then mounted onto object glass using an anti-fade mounting agent containing DAPI. The samples were imaged under a fluorescence microscope (Olympus, Shinjuku City, Japan).

### 2.8. Statistical Analysis

Quantitative data are presented as the means ± standard deviation. Differences were compared using Student’s *t*-test. *p*-values less than 0.05 were considered statistically significant.

## 3. Results

### 3.1. Synthesis of Lipidated Pepstatin A

To efficiently link pepstatin A to the liposomal surface, we conjugated the lipid tail with the peptide part through a long polyethylene glycol-2000 (PEG(2000)) linker. This was achieved by joining a DSPE-PEG(2000) amine to pepstatin A through an amide coupling reaction (Figure 1a). The reaction’s progress was monitored using LC-MS; the yield was 10 mg, or 59% of the theoretical yield. The lipid tail of the lipidated pepstatin A thus enabled its insertion into the lipid bilayer, while the PEG(2000) linker ensured that the peptide part was accessible to cathepsin D.

### 3.2. Pepstatin A Inhibition of Cathepsin D87

**Figure 2:**
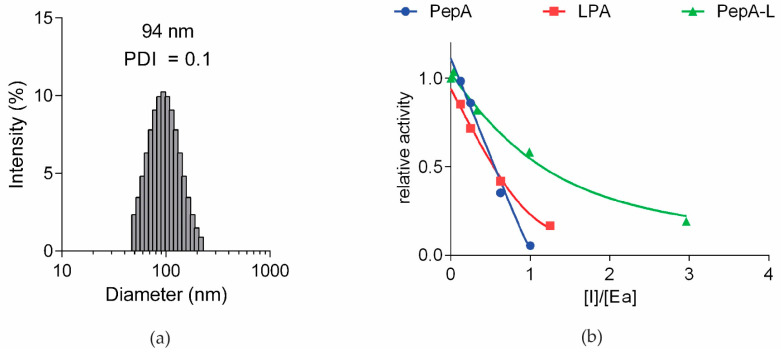
(**a**) DLS measurement of PepA-L, showing average diameter and size distribution. (**b**) Inhibition of recombinant cathepsin D activity. Relative activity (ratio of inhibited vs. uninhibited enzyme activity) was determined in the presence of increasing concentrations of pepstatin A (PepA), lipidated PepA (LPA), or pepstatin A-liposomes (PepA-L).

The synthesized LPA was incorporated into a liposomal lipid bilayer using the extrusion method, producing pepstatin-A-functionalized liposomes with an average diameter of 94 nm and a PDI = 0.1 as measured using dynamic light scattering (DLS) (Figure 2a). To confirm that adding the lipid tail or insertion into the surface of liposomes did not interfere with binding and inhibitory activity, both the LPA and PepA-L were tested for Cathepsin D inhibition compared to non-conjugated pepstatin A (PepA) (Figure 2b). All three compounds successfully inhibited recombinant cathepsin D activity, with LPA showing comparable inhibition to non-conjugated pepstatin A. Therefore, the addition of a lipid tail with a linker did not interfere with the inhibitory properties of pepstatin A. PepA-L inhibited cathepsin D activity slightly less efficiently, which could be attributed to steric hindrances imposed on the inhibitory molecule via its binding on the surface of the liposome. Furthermore, a fraction of pepstatin A could be localized in the inner membrane of the liposomes if a small fraction of multilamellar vesicles also formed during extrusion, thus further explaining the reduction in cathepsin D inhibition induced by PepA-L.

### 3.3. Cathepsin D Expression in Breast Cancer Cell Lines

**Figure 3:**
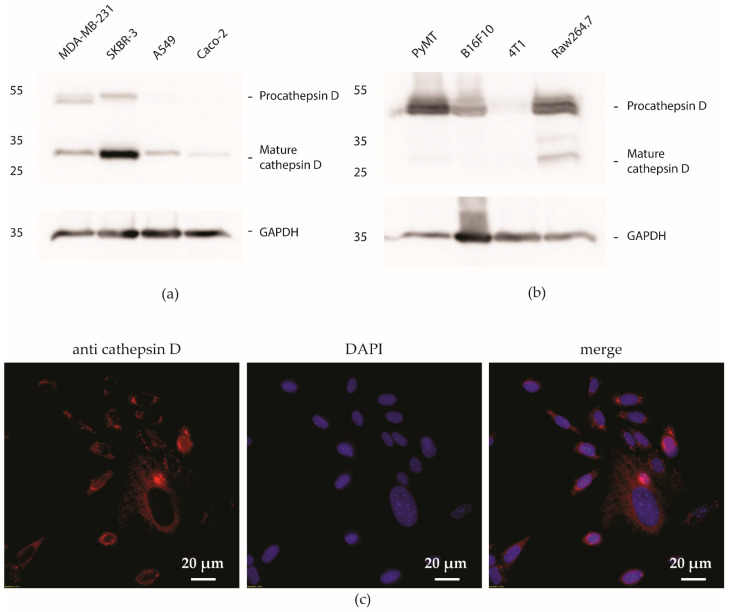
Detecting the presence of procathepsin D and mature cathepsin D using immunoblot assay in four different human (**a**) and four murine cell lines (**b**). Immunocytochemistry of PyMT cells (**c**), labelled with anti-cathepsin D antibodies and the nuclear dye DAPI, confirmed the presence of cathepsin D in cells.

We first screened for the presence of cathepsin D in several human and murine cell lines. The human cell lines included A549, CaCO-2, MDA-MB-231, and SK-BR-3. The murine cells included 4T1, B16F10, PyMT, and RAW 264.7. Lysates from these cells were then immunoblotted against cathepsin D (Figure 3a,b). We were able to detect both the proenzyme and active form of the protease in human cell lines and murine cells at molecular sizes of around 30 kDa and 50 kDa, respectively. The signal band quantification data are included in the supplementary file (Figure S2a) as well as whole-membrane images (Figure S2b,c). Notably, the active form of cathepsin D was more prominent in human cell lines, while the proenzyme counterpart was more abundant in murine cells. Cathepsin D is heavily expressed in breast cancer [14,17,35]. In agreement with this, cathepsin D expression was high in both breast cancer cell lines, i.e., MDA-MB-231 and SK-BR-3, with the signal of active cathepsin D being highest in SK-BR-3. Cathepsin D expression was low in the lung-adenocarcinoma-derived cell line (A549) and the colon-carcinoma-derived cell line (CaCO-2). In murine cells, cathepsin D expression was the highest in primary cells from PyMT mice and in the macrophage-like cell line (RAW 264.7). The expression of cathepsin D in the melanoma cell line (B16F10) was only slightly lower than that in the PyMT or RAW 264.7 cells, and we detected almost no expression of cathepsin D in the mammary carcinoma cell line (4T1). The signal for active cathepsin D was present only in the RAW 264.7 cell line. The two human cell lines, MDA-MB-231 and SK-BR-3, and two murine cell lines, RAW 264.7 and PyMT, with the highest cathepsin D expression, were used in cell-binding assays. Additionally, we confirmed the presence of cathepsin D in the PyMT cells by employing immunocytochemistry (Figure 3c).

### 3.4. Pepstatin A Liposomes Binding to Cells

**Figure 4:**
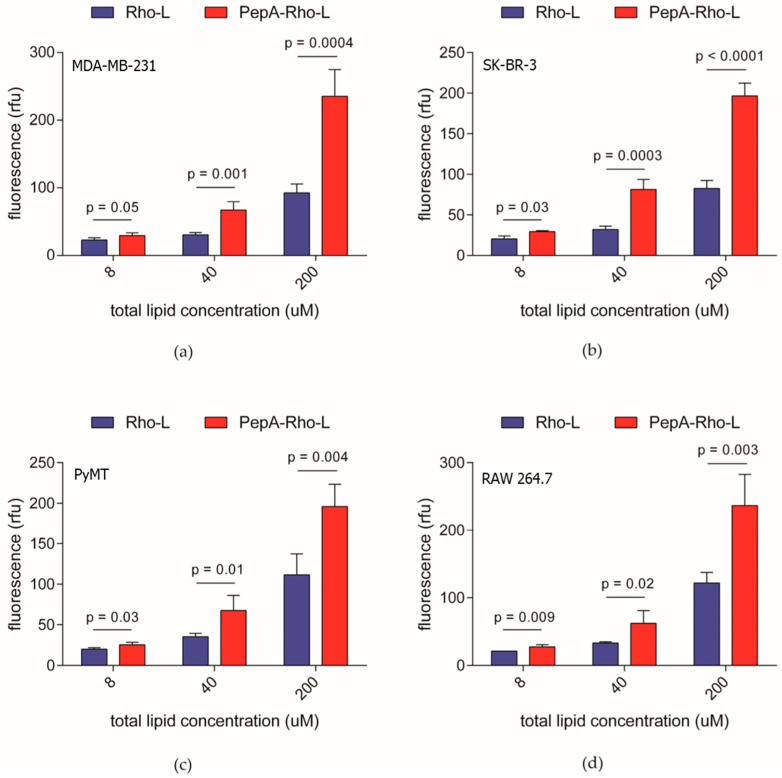
Two human cell lines and two murine cell lines were used in cell association assay. The images correspond to fluorescence intensity (l ex/em = 560/580 nm) in washed MDA-MB-231 (**a**), SK-BR-3 (**b**), PyMT (**c**), or RAW 264.7 (**d**) cells following incubation with increasing concentrations of non-functionalized rhodamine-labeled liposomes (Rho-L) or pepstatin A-rhodamine-labeled liposomes (PepA-Rho-L), *n* = 4. Data are presented as means ± standard deviation.

Next, we tested the binding of PepA-L to the surface of tumor or tumor-associated cells. To target only the membrane-bound extracellular cathepsin D, we blocked endocytosis by performing the binding experiment at 4 °C. For this study, liposomes were loaded with rhodamine B to enable their detection via measuring fluorescence. Cells were then incubated with increasing concentrations of PepA-L or nonconjugated liposomes, and fluorescence was measured after washing the cells. In all cell lines, the fluorescence signal increased with the increasing concentration of both pepstatin A-conjugated and non-conjugated liposomes (Figure 4a–d). However, the signal from the pepstatin A-conjugated liposomes was significantly higher than that from the non-conjugated liposomes in all the cell lines tested. The fluorescence signal intensity as well as the difference between the signal from PepA-L and the non-conjugated liposomes were comparable among all cell lines despite the differences in the expression level of active cathepsin D. We also tested the binding of PepA-L to the 4T1 murine cell surface: there was no significant difference in fluorescence between the Rho-L- and PepA-Rho-L-treated cells (Figure S3).

In the final step, we investigated the therapeutic applicability of the PepA-L targeted drug delivery system. We performed an in vitro proliferation study on PyMT cells, which were treated with pepstatin-A-functionalized doxorubicin-loaded liposomes (PepA-L-Dox), non-functionalized doxorubicin-loaded liposomes (L-Dox), or pepstatin-A-functionalized liposomes without doxorubicin (PepA-L) (Figure S1). The effect of PepA-L-Dox on the proliferation was greater compared to the effect of L-Dox, and the difference was more pronounced at lower concentrations of doxorubicin.

## 4. Discussion

Proteases are often overexpressed and overly active during cancer progression, making them potential targets in cancer therapy [36,37,38]. Unlike other proteases, such as MMPs, which are present extracellularly in normal tissue, cathepsins are only excreted into the extracellular space in pathological conditions [7,39]. Therefore, targeting cathepsins represents a potentially safer strategy and can reduce toxicity risk for healthy tissues. Several researchers in the tumor-targeting-drug field have focused on targeting cysteine cathepsins, while the aspartic cathepsin family has been mainly neglected [40]. Cathepsin D is an aspartic protease overexpressed in many different cancer types. Its roles in several stages of cancer development have been demonstrated, and it has been considered a promising therapeutic target [11,13,15,38]. In a recent study by Ashraf et al., cathepsin-D-targeting antibodies were employed in the treatment of triple-negative breast cancer. Using a mouse xenograft tumor model, the authors demonstrated that the application of human anti-cathepsin D antibodies inhibited tumor growth by preventing macrophage recruitment and triggered natural killer cell activation [41]. Contrastingly, cathepsin D could also be used as an efficient cancer drug delivery target due to its abundance in the TME.

Here, we report the development of a liposome-based targeting system based on pepstatin A as a selective moiety for targeting cathepsin D. Utilizing the potent peptide inhibitor of aspartic proteases, pepstatin A, as a targeting moiety has several advantages. First, pepstatin A binds to its target—cathepsin D—with a dissociation constant (KD) of around 0.4 nM [42], surpassing antibodies and small molecules in this regard. Second, as a small peptide, pepstatin A is more stable and robust, making it a practical and cost-effective targeting moiety. This idea was confirmed by demonstrating that neither lipidation nor liposomal insertion substantially affected its inhibitory properties (Figure 2b).

Liposomal targeting systems utilizing protease inhibitors have been previously developed for cathepsin B [9] and cathepsins S and L [8], wherein the targeting moieties were a small molecule or a small protein inhibitor, respectively. Both systems successfully targeted the TME in murine cancer models, demonstrated by their efficient accumulation at the tumor site. As described in our study, the cathepsin D targeting system demonstrated successful binding to tumor cells in vitro, suggesting that it could similarly accumulate in the TME in vivo. Equipped with magnetic resonance contrast agents, pepstatin A-functionalized liposomes could be used as diagnostic tools in a wide array of cancers overexpressing cathepsin D. Moreover, liposomes could be loaded with a chemotherapeutic agent and thus utilized as a potent drug delivery system. However, one of the potential disadvantages of liposomes could be immunogenicity, which should be tested for each individual lipid nanoparticle [43].

In conclusion, our newly developed targeting system based on liposome-bound pepstatin A successfully inhibited cathepsin D and demonstrated efficient binding to the tumor cell surface. Pepstatin-A-functionalized liposomes could be successfully utilized in diagnostics, taking advantage of the significant overexpression of cathepsin D in various types of cancer. Additionally, combined with chemotherapeutic agents, the system could be used for drug delivery or theragnostic applications.

## Data Availability

Not applicable.

## Supplementary Materials

The following supporting information can be downloaded at: https://www.mdpi.com/article/10.3390/pharmaceutics15102464/s1, Figure S1: Effect of doxorubicin loaded liposomes on proliferation of PyMT cells; Figure S2: (a) Normalized expression of procathepsin D and mature cathepsin D in different cell lines. (b) Whole western blot membrane image with anti-cathepsin D antibodies. (c) Whole western blot membrane image with anti-GAPDH antibodies; Figure S3: Cell association assay for the 4t1 murine cell line; Table S1: Cell association assay fluorescent signals.

## References

[1] Lee Y.T., Tan Y.J., Oon C.E. (2018). Molecular targeted therapy: Treating cancer with specificity. Eur. J. Pharmacol..

[2] Pérez-Herrero E., Fernández-Medarde A. (2015). Advanced targeted therapies in cancer: Drug nanocarriers, the future of chemotherapy. Eur. J. Pharm. Biopharm..

[3] Vasiljeva O., Hostetter D.R., Moore S.J., Winter M.B. (2019). The multifaceted roles of tumor-associated proteases and harnessing their activity for prodrug activation. Biol. Chem..

[4] Hanahan D., Weinberg R.A. (2011). Hallmarks of cancer: The next generation. Cell.

[5] Balkwill F.R., Capasso M., Hagemann T. (2012). The tumor microenvironment at a glance. J. Cell Sci..

[6] Olson O.C., Joyce J.A. (2015). Cysteine cathepsin proteases: Regulators of cancer progression and therapeutic response. Nat. Rev. Cancer.

[7] Vidak E., Javoršek U., Vizovišek M., Turk B. (2019). Cysteine Cathepsins and Their Extracellular Roles: Shaping the Microenvironment. Cells.

[8] Bratovš A., Kramer L., Mikhaylov G., Vasiljeva O., Turk B. (2019). Stefin A-functionalized liposomes as a system for cathepsins S and L-targeted drug delivery. Biochimie.

[9] Mikhaylov G., Klimpel D., Schaschke N., Mikac U., Vizovisek M., Fonovic M., Turk V., Turk B., Vasiljeva O. (2014). Selective Targeting of Tumor and Stromal Cells By a Nanocarrier System Displaying Lipidated Cathepsin B Inhibitor. Angew. Chem. Int. Ed..

[10] Kramer L., Renko M., Završnik J., Turk D., Seeger M.A., Vasiljeva O., Grütter M.G., Turk V., Turk B. (2017). Non-invasive *in vivo* imaging of tumour-associated cathepsin B by a highly selective inhibitory DARPin. Theranostics.

[11] Dubey V., Luqman S. (2017). Cathepsin D as a Promising Target for the Discovery of Novel Anticancer Agents. Curr. Cancer Drug Targets.

[12] Benes P., Vetvicka V., Fusek M. (2008). Cathepsin D—Many functions of one aspartic protease. Crit. Rev. Oncol. Hematol..

[13] Liaudet-Coopman E., Beaujouin M., Derocq D., Garcia M., Glondu-Lassis M., Laurent-Matha V., Prébois C., Rochefort H., Vignon F. (2006). Cathepsin D: Newly discovered functions of a long-standing aspartic protease in cancer and apoptosis. Cancer Lett..

[14] Rochefort H. (1990). Cathepsin D in breast cancer. Breast Cancer Res. Treat..

[15] Berchem G., Glondu M., Gleizes M., Brouillet J.-P., Vignon F., Garcia M., Liaudet-Coopman E. (2002). Cathepsin-D affects multiple tumor progression steps in vivo: Proliferation, angiogenesis and apoptosis. Oncogene.

[16] Pranjol Z.I., Gutowski N.J., Hannemann M., Whatmore J.L. (2018). Cathepsin D non-proteolytically induces proliferation and migration in human omental microvascular endothelial cells via activation of the ERK1/2 and PI3K/AKT pathways. Biochim. Biophys. Acta (BBA)-Mol. Cell Res..

[17] Pranjol Z.I., Whatmore J.L. (2020). Cathepsin D in the Tumor Microenvironment of Breast and Ovarian Cancers. Adv. Exp. Med. Biol..

[18] Briozzo P., Morisset M., Capony F., Rougeot C., Rochefort H. (1988). In vitro degradation of extracellular matrix with Mr 52,000 cathepsin D secreted by breast cancer cells. Cancer Res..

[19] Laurent-Matha V., Maruani-Herrmann S., Prébois C., Beaujouin M., Glondu M., Noël A., Alvarez-Gonzalez M.L., Blacher S., Coopman P., Baghdiguian S. (2005). Catalytically inactive human cathepsin D triggers fibroblast invasive growth. J. Cell Biol..

[20] Allen T.M., Cullis P.R. (2013). Liposomal drug delivery systems: From concept to clinical applications. Adv. Drug Deliv. Rev..

[21] Tran S., DeGiovanni P.-J., Piel B., Rai P. (2017). Cancer nanomedicine: A review of recent success in drug delivery. Clin. Transl. Med..

[22] Mikhaylov G., Mikac U., Magaeva A.A., Itin V.I., Naiden E.P., Psakhye I., Babes L., Reinheckel T., Peters C., Zeiser R. (2011). Ferri-liposomes as an MRI-visible drug-delivery system for targeting tumours and their microenvironment. Nat. Nanotechnol..

[23] Deshpande P.P., Biswas S., Torchilin V.P. (2013). Current trends in the use of liposomes for tumor targeting. Nanomedicine.

[24] Gindy M.E., Prud’Homme R.K. (2009). Multifunctional nanoparticles for imaging, delivery and targeting in cancer therapy. Expert Opin. Drug Deliv..

[25] Kraft J.C., Freeling J.P., Wang Z., Ho R.J. (2014). Emerging research and clinical development trends of liposome and lipid nanoparticle drug delivery systems. J. Pharm. Sci..

[26] Schoenmaker L., Witzigmann D., Kulkarni J.A., Verbeke R., Kersten G., Jiskoot W., Crommelin D.J. (2021). mRNA-lipid nanoparticle COVID-19 vaccines: Structure and stability. Int. J. Pharm..

[27] Barenholz Y. (2012). (Chezy) Doxil^®^—The first FDA-approved nano-drug: Lessons learned. J. Control. Release.

[28] Baden L.R., El Sahly H.M., Essink B., Kotloff K., Frey S., Novak R., Diemert D., Spector S.A., Rouphael N., Creech C.B. (2021). Efficacy and Safety of the mRNA-1273 SARS-CoV-2 Vaccine. N. Engl. J. Med..

[29] Polack F.P., Thomas S.J., Kitchin N., Absalon J., Gurtman A., Lockhart S., Perez J.L., Pérez Marc G., Moreira E.D., Zerbini C. (2020). Safety and Efficacy of the BNT162b2 mRNA COVID-19 Vaccine. N. Engl. J. Med..

[30] Tenchov R., Bird R., Curtze A.E., Zhou Q. (2021). Lipid Nanoparticles─From Liposomes to mRNA Vaccine Delivery, a Landscape of Research Diversity and Advancement. ACS Nano.

[31] Umezawa H., Aoyagi T., Morishima H., Matsuzaki M., Hamada M. (1970). Pepstatin, a new pepsin inhibitor produced by Actinomycetes. J. Antibiot..

[32] Baldwin E.T., Bhat T.N., Gulnik S., Hosur M.V., Sowder R.C., Cachau R.E., Collins J., Silva A.M., Erickson J.W. (1993). Crystal structures of native and inhibited forms of human cathepsin D: Implications for lysosomal targeting and drug design. Proc. Natl. Acad. Sci. USA.

[33] Vasiljeva O., Papazoglou A., Krüger A., Brodoefel H., Korovin M., Deussing J., Augustin N., Nielsen B.S., Almholt K., Bogyo M. (2006). Tumor cell–derived and macrophage-derived cathepsin B promotes progression and lung metastasis of mammary cancer. Cancer Res..

[34] Kopitar-Jerala N., Puizdar V., Berbić S., Zavašnik–Bergant T., Turk V. (2001). A cathepsin D specific monoclonal antibody. Immunol. Lett..

[35] Garcia M., Platet N., Liaudet E., Laurent V., Derocq D., Brouillet J., Rochefort H. (1996). Biological and clinical significance of cathepsin d in breast cancer metastasis. Stem Cells.

[36] Abbenante G., Fairlie D.P. (2005). Protease inhibitors in the clinic. Med. Chem..

[37] Turk B. (2006). Targeting proteases: Successes, failures and future prospects. Nat. Rev. Drug Discov..

[38] Pranjol Z.I., Whatmore J.L., Birbrair A. (2020). Cathepsin D in the Tumor Microenvironment of Breast and Ovarian Cancers in Tumor Microenvironment: Molecular Players—Part A.

[39] Turk V., Stoka V., Vasiljeva O., Renko M., Sun T., Turk B., Turk D. (2012). Cysteine cathepsins: From structure, function and regulation to new frontiers. Biochim. Biophys. Acta BBA-Proteins Proteom..

[40] Kramer L., Turk D., Turk B. (2017). The Future of Cysteine Cathepsins in Disease Management. Trends Pharmacol. Sci..

[41] Ashraf Y., Mansouri H., Laurent-Matha V., Alcaraz L.B., Roger P., Guiu S., Derocq D., Robin G., Michaud H.-A., Delpech H. (2019). Immunotherapy of triple-negative breast cancer with cathepsin D-targeting antibodies. J. Immunother. Cancer.

[42] Knight C.G., Barrett A.J. (1976). Interaction of human cathepsin D with the inhibitor pepstatin. Biochem. J..

[43] Kiaie S.H., Majidi Zolbanin N., Ahmadi A., Bagherifar R., Valizadeh H., Kashanchi F., Jafari R. (2022). Recent advances in mRNA-LNP therapeutics: Immunological and pharmacological aspects. J. Nanobiotechnology.

