# Supplementary material for: A New Cathepsin D Targeting Drug Delivery System Based on Immunoliposomes Functionalized with Lipidated Pepstatin A
Source: Pharmaceutics. 2023 Oct 14;15(10):2464. doi: 10.3390/pharmaceutics15102464 (PMC10609775; doi:10.3390/pharmaceutics15102464)
Supplement: Supplementary file 1 [file pharmaceutics-15-02464-s001.zip › pharmaceutics-2215410-supplementary.pdf]

A New Cathepsin D Targeting Drug Delivery System Based on Immunoliposomes Functionalized with Lipidated Pepstatin A

Andreja Kozak [1], Georgy Mikhaylov [1,2], Pavlo Khodakivskyi [3], Elena Goun [2,3], Boris Turk [1,4,*] and Olga Vasiljeva [1,*,†]

Supplementary Figures

(a)

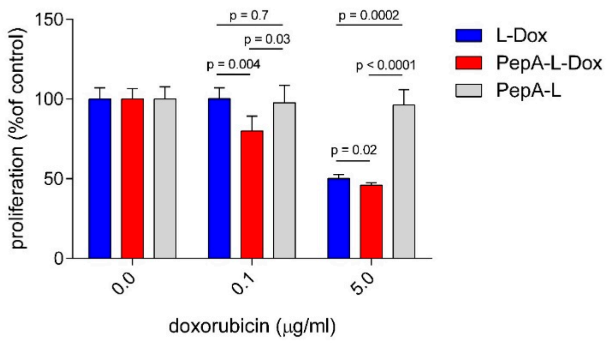

(b)

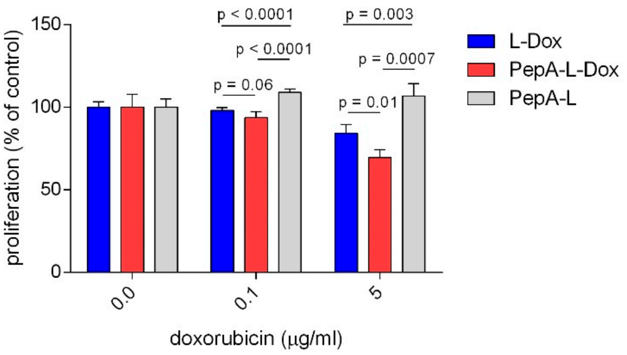

(c)

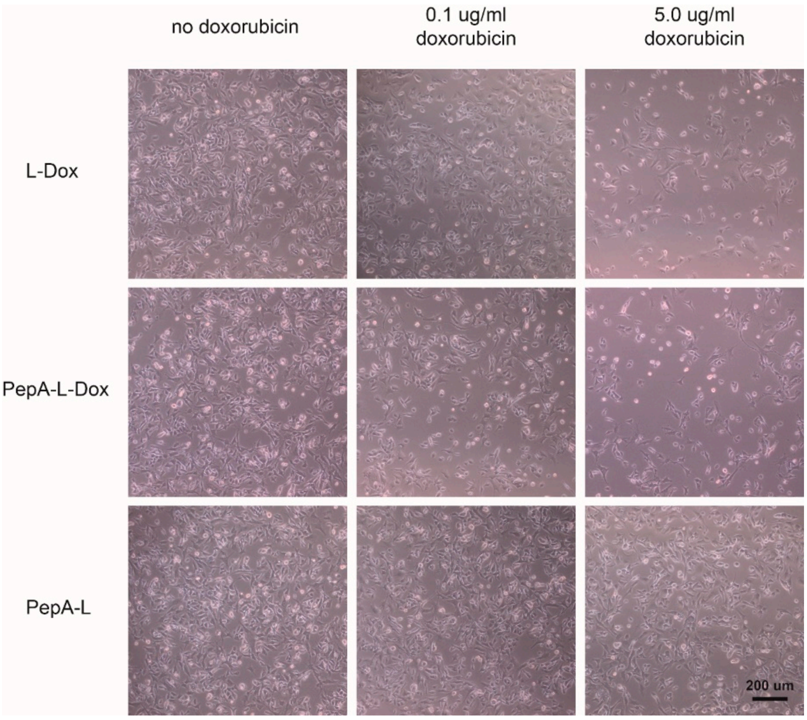

Figure S1. Effect of doxorubicin loaded liposomes on proliferation of PyMT cells. PyMT cells were incubated with non-functionalized doxorubicin loaded liposomes (L-Dox), pepstatin A-functionalized doxorubicin loaded liposomes (PepA-L-Dox) or pepstatin A-functionalized liposomes without doxorubicin (PepA-L). After 24h, proliferation was measured using the BrdU assay (a) or MTT assay (b). **(c)** Microscopic images of cells 24h after treatment with liposomes. Quantitative data is presented as the mean ± standard deviation.

(a)

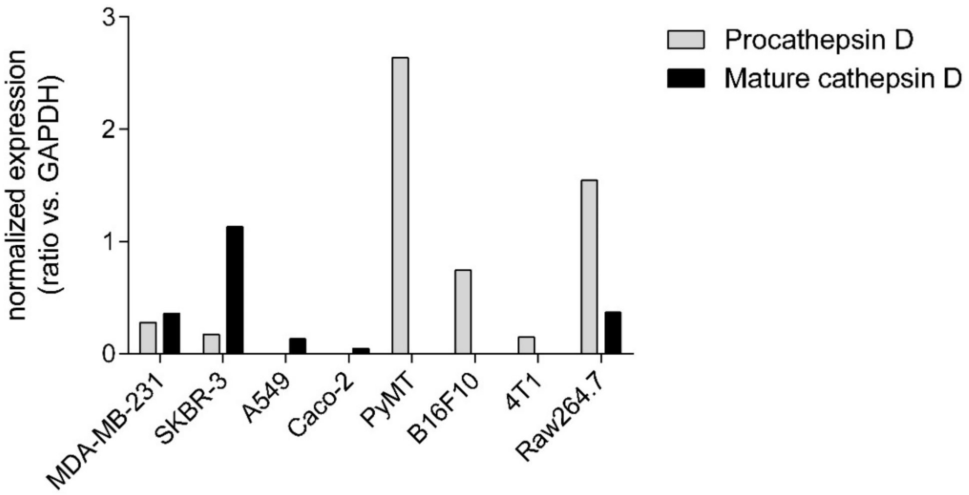

(b)

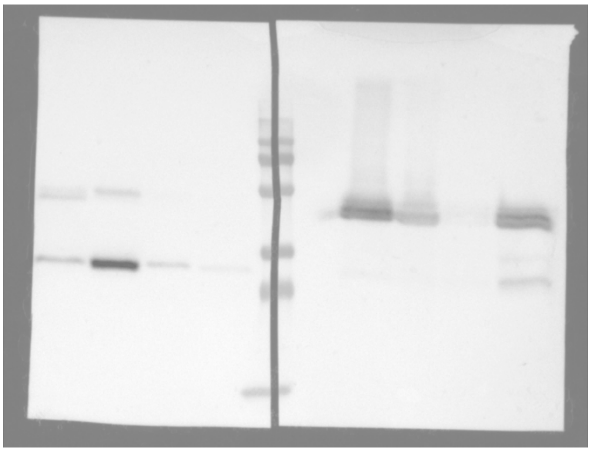

(c)

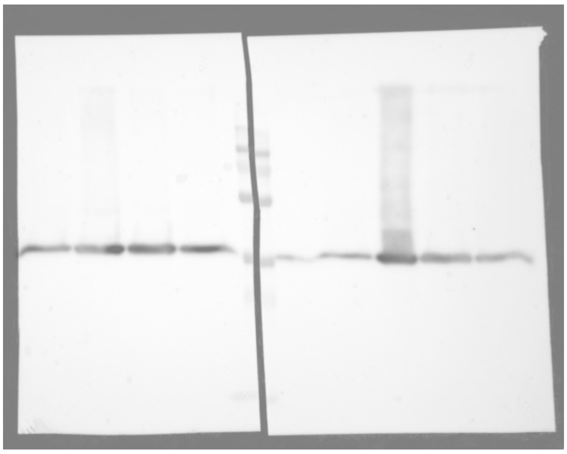

Figure S2. (a) Normalized expression of procathepsin D and mature cathepsin D in different cell lines. Signal bands were quantified using GeneTools software (Synoptics Ltd.) and are presented as ratios of pro- or mature cathepsin D and GADPH. **(b)** Whole western blot membrane image with anti-cathepsin D antibodies. **(c)** Whole western blot membrane image with anti-GAPDH antibodies.

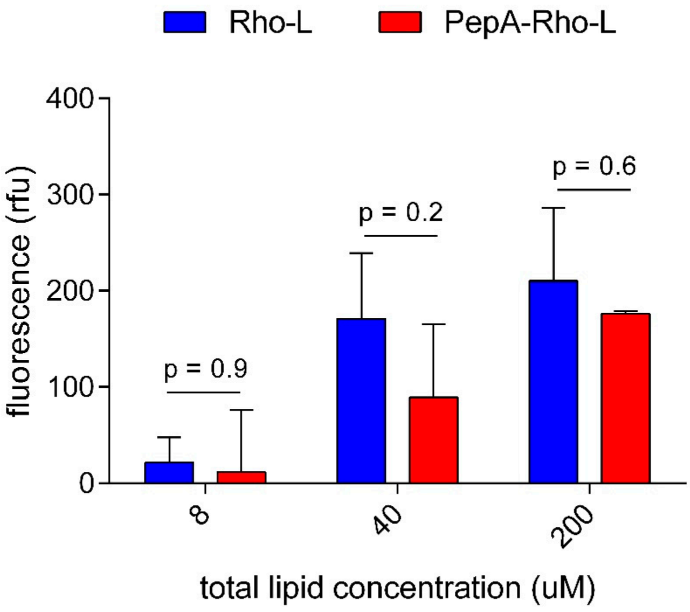

Figure S3. Cell association assay for the 4t1 murine cell line. Fluorescence intensity (l ex/em = 560/580 nm) in washed cells following incubation with increasing concentration of non-functionalized rhodamine-labeled liposomes (Rho-L) or pepstatin A-rhodamine-labeled liposomes (PepA-Rho-L), n = 3.

Table S1. Cell association assay fluorescent signals. Fluorescence intensity (l ex/em = 560/580 nm, RFU) in washed MDA-MB-231 **(a)**, SK-BR-3 **(b)**, PyMT **(c)**, or RAW 264.7 **(d)** cells following incubation with increasing concentration of non-functionalized rhodamine-labeled liposomes (Rho-L) or pepstatin A-rhodamine-labeled liposomes (PepA-Rho-L), n = 4.

**Cell line**	**Liposomes**	**Total lipid concentration**		
		**8 µM**	**40 µM**	**200 µM**
MDA-MB-231	Rho-L	23 ± 3	30 ± 3	93 ± 13
	PepA-Rho-L	29 ± 4	67 ± 13	235 ± 40
SK-BR-3	Rho-L	20 ± 4	32 ± 4	83 ± 10
	PepA-Rho-L	30 ± 1	81 ± 12	197 ± 16
PyMT	Rho-L	20 ± 1	35 ± 4	112 ± 26
	PepA-Rho-L	25 ± 3	68 ± 19	196 ± 27
RAW 264.7	Rho-L	21 ± 0	33 ± 2	122 ± 16
	PepA-Rho-L	27 ± 3	62 ± 19	237 ± 46

Supplementary Method

Preparation of doxorubicin-loaded liposomes

Liposomes were prepared from chicken egg L-a-phosphatidylcholine (63 mol %) (Avanti Polar Lipids), cholesterol (33 mol %) (Sigma), and MeO-PEG (4 mol %) (Avanti Polar Lipids). For pepstatin A functionalized liposomes (PepA-L), methoxy-PEG was re-placed with lipidated PEG-pepstatin A (LPA). The total lipid concentration was 10 mM. The organic solvent was evaporated in an Eppendorf Concentrator 5301 (Eppendorf). The dry lipid film formed was then hydrated in degassed 300 mM ammonium sulfate buffer (pH 5.0). To generate nanosized unilamellar bilayer liposomes, the

multilamellar formed vesicles were extruded using a mini-extruder (Avanti Polar Lipids), fitted with a polycarbonate membrane, pore size 400 nm, followed by extrusion through a 100 nm pore size membrane. Liposomes were then loaded on a PD-10 desalting column (Cytiva) to replace the outside liposoma buffer with 100 mM sodium phosphate buffer (pH 7.0, 150 mM NaCl). Doxorubicin-HCl (LC Laboratories) was added to the liposomes to a final concentration of 150 μg/mL. Liposomes were then gently shaken at 4 °C overnight to yield non-functionalized doxorubicin loaded liposomes (L-Dox) or pepstatin A-functionalized doxorubicin loaded liposomes (PepA-L-Dox).

Cell proliferation assay

Cells (10,000 per well) were seeded into white 96-well plates with transparent bottoms (Falcon) and grown overnight at 37 °C and 5 % CO_2. Cells were then incubated at 4 °C for 30 min to block the endocytosis process; the following steps were also performed at 4 °C. Non-functionalized doxorubicin loaded liposomes (L-Dox), pepstatin A-functionalized doxorubicin loaded liposomes (PepA-L-Dox) or pepstatin A-functionalized liposomes without doxorubicin (PepA-L) were added to the cells at increasing concentrations. Cells were then incubated with the liposomes for 3h at 4 °C. Next, they were washed with PBS two times and fresh cDMEM was added. Cells were then grown for 24h at 37 °C and 5 % CO_2. After that, BrdU chemiluminescent assay (Abcam) was performed according to the manufactorer's instructions. Luminescence was measured in the IVIS system (Perkin Elmer). For the MTT assay, cells were seeded into transparent 96-well plates instead, then treated as described above. After the 24h growth in fresh cDMEM, MTT reagent was added to a final concentration of 0.5 mg/mL. cells were then incubated at 37 °C and 5 % CO_2 for 2.5h, then DMSO was added for solubilization of crystals and absorbance was measured at 570 nm using the plate reader (TECAN).
